# Innate Immunity: A Common Denominator between Neurodegenerative and Neuropsychiatric Diseases

**DOI:** 10.3390/ijms21031115

**Published:** 2020-02-07

**Authors:** Fabiana Novellino, Valeria Saccà, Annalidia Donato, Paolo Zaffino, Maria Francesca Spadea, Marco Vismara, Biagio Arcidiacono, Natalia Malara, Ivan Presta, Giuseppe Donato

**Affiliations:** 1 Neuroimaging Unit, Institute of Bioimaging and Molecular Physiology, National Research Council (IBFM-CNR) Viale Europa, 88100 Catanzaro, Italy; 2 Department of Medical and Surgical Sciences, University “Magna Graecia” of Catanzaro, 88100 Catanzaro, Italy; valeria.sacca87@gmail.com (V.S.); annalidia.donato@gmail.com (A.D.); 3 Department of Clinical and Experimental Medicine, University “Magna Graecia” of Catanzaro, 88100 Catanzaro, Italy; p.zaffino@unicz.it (P.Z.); mfspadea@unicz.it (M.F.S.); nataliamalara@unicz.it (N.M.); 4 Department of Cell Biotechnologies and Hematology, University “La Sapienza” of Rome, 00185 Rome, Italy; marco.vismara@uniroma1.it; 5 Department of Health Sciences, University “Magna Græcia” of Catanzaro, 88100 Catanzaro, Italy; arcidiacono@unicz.it (B.A.); presta@unicz.it (I.P.); gdonato@unicz.it (G.D.)

**Keywords:** innate immunity, neurodegeneration, microbiota-immune axis, Parkinson’s disease, Huntington’s disease, amyotrophic lateral sclerosis, frontotemporal dementia, depressive disorders, schizophrenia, autism spectrum disorder

## Abstract

The intricate relationships between innate immunity and brain diseases raise increased interest across the wide spectrum of neurodegenerative and neuropsychiatric disorders. Barriers, such as the blood–brain barrier, and innate immunity cells such as microglia, astrocytes, macrophages, and mast cells are involved in triggering disease events in these groups, through the action of many different cytokines. Chronic inflammation can lead to dysfunctions in large-scale brain networks. Neurodegenerative diseases, such as Alzheimer’s disease, Parkinson’s disease, Huntington’s disease, amyotrophic lateral sclerosis, and frontotemporal dementia, are associated with a substrate of dysregulated immune responses that impair the central nervous system balance. Recent evidence suggests that similar phenomena are involved in psychiatric diseases, such as depression, schizophrenia, autism spectrum disorders, and post-traumatic stress disorder. The present review summarizes and discusses the main evidence linking the innate immunological response in neurodegenerative and psychiatric diseases, thus providing insights into how the responses of innate immunity represent a common denominator between diseases belonging to the neurological and psychiatric sphere. Improved knowledge of such immunological aspects could provide the framework for the future development of new diagnostic and therapeutic approaches.

## 1. Introduction

Innate immunity acts via both non-inducible and inducible mechanisms. The body has natural anatomical and physiological barriers that act non-specifically to prevent infections. If these barriers are crossed, patterns of recognition that are mediated either by soluble molecules secreted in the extracellular space or by receptors expressed on the surface of innate leucocytes trigger inducible innate mechanisms. The blood–brain barrier (BBB) is not strictly recognized as an anatomical barrier of the innate immunity system, however its crosstalking with the cells of the innate immunity (such as macrophage, mast cells, and polymorphonuclear leucocytes) is increasingly viewed as fundamental in the regulation of the immune state of the central nervous system (CNS). 

**Figure 1:**
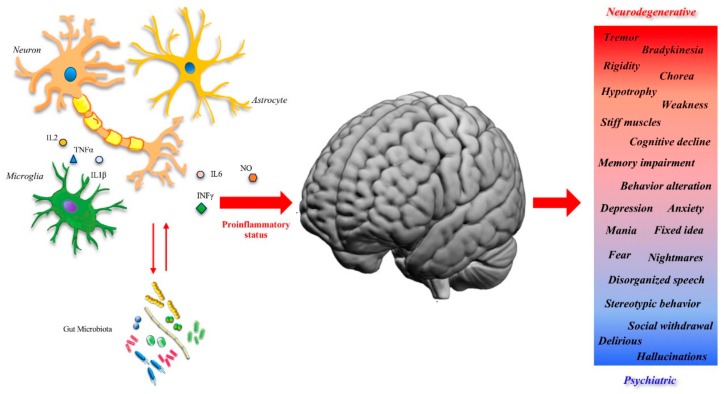
Innate immunity dysregulation is a pathophysiological mechanism shared between neurological and psychiatric brain diseases. As common substrates become clear, the exact distinction between these disorders becomes nuanced.

Emerging evidence suggests that innate immunity produces resistance by sustaining reactivity to pathogenic agents and by sustaining non-specific long-term responses. Although the innate immunity action usually plays a beneficial role in host defense, the current literature shows that sometimes it has a negative impact. Indeed, under predisposing conditions, the innate immunity may contribute to human diseases characterized by an uncontrolled increase in inflammation, even sustaining a neuroinflammatory cycle [1,2]. Innate immunity plays a pivotal role in the growth and progression of brain tumors and in various neurological diseases [3,4]. Dysregulated innate immune cells and uncontrolled neuroinflammatory processes appear to be critical in several neurodegenerative and psychiatric diseases (Figure 1). However, it is still not clear exactly how the innate immunity response influences pathogenetic phenomena. 

The aim of this review is to highlight the substantial evidence of the role of innate immunity in the pathophysiology of neurodegenerative and psychiatric diseases. The unregulated response of innate immunity represents a common denominator between diseases belonging to the neurological and psychiatric framework. 

## 2. Neurodegenerative Diseases

### 2.1. Alzheimer’s Disease

Alzheimer’s disease (AD) is an inexorably progressive and irreversible brain disorder that affects higher cognitive functions. Memory loss is the typical sign of the disease, but there is also a significant decline in other domains of cognition (language, visual–spatial skills, praxic skills, reasoning, and judgment capability). AD is the most frequent cause of cognitive decline in senile age [5], with very high medical, social, and financial costs. Despite the great efforts to identify the causes of AD, the pathogenetic mechanisms have not been fully clarified and there are no effective therapies to stop or reverse its progression. The accumulation of abnormally folded amyloid-beta (senile plaques) and tau (neurofibrillary tangles) proteins is the distinctive pathological hallmark of the disease [6]. Changes primarily occur in the enthorhinal cortex and hippocampus, and then spread in the brain. [7,8,9].

Pathological and biochemical studies have highlighted the presence of immune-associated compounds in AD brain parenchyma, especially in proximity to the senile plaques and neurofibrillary tangles. This includes (but is not limited to) complement components, proinflammatory interleukins (IL-1 and IL-6), macrophage colony-stimulating factor (M-CSF), transforming growth factor β (TGFβ), tumor necrosis factor α (TNFα), and C-reactive protein (CRP) [10]. In addition, immunological infiltrates within the brain have been recognized in AD, mainly belonging to the innate immunity arm (i.e., resident microglia, peripheral monocytes/macrophages) [11]. The presence of activated microglia has been demonstrated in vivo through positron emission tomography (PET) imaging studies. The cerebral binding of the PK11195 (1-(2-chlorophenyl)-N-methyl-N-1(1-methylpropyl)-3-isoquinolinecarboxamide) ligand (considered a marker of microglial activation) has been found to be enhanced in AD patients and correlated with disease severity [12,13,14]. Therefore, the role of inflammation in AD, and in particular of the innate immunity, has been increasingly studied. 

Our group recently reviewed various immunological mechanisms involved in AD [4], highlighting the role of microglia and chronic low-grade inflammatory stimuli, which produce different levels of immune dysregulation depending on the stage of the disease. Neuroinflammation is not simply a reactive response activated by incoming senile plaques and neurofibrillary tangles, but instead contributes as much to pathogenesis as do plaques and tangles [15]. In fact, the innate immunity response in AD is complex, with multiple and extremely disease-stage-specific effects in AD pathophysiology. 

In pre-symptomatic and early AD, the immune response limits amyloid-beta plaque formation; however, the repeated triggering of microglial cells, due to the ongoing production of proinflammatory cytokines, causes a microglia detrimental trained potentiation, which contributes to disease progression. Lastly, at advanced stages of AD, high exposure to continous stimulus causes a microglia phenotypic switch into a dysfunctional senescent state with weak phagocytosis and a tolerance state characterized by the decreased release of proinflammatory compounds [16].

Recent observations suggest that, besides the central immune response, peripheral inflammation also plays a crucial role in AD [17,18]. Microglia are responsive to proinflammatory mediators produced outside the cerebral compartment, and peripheral inflammation, together with the recruitment of blood-borne innate immune cells, is decisive for disease progression [19]. Therefore, when considering the innate immunity participation in the pathogenic cascade of AD, the close interaction between brain resident and circulating innate immune myeloid cells should be considered. The myeloid cells work both direct (by infiltrating into the brain parenchyma) and indirect effects (by releasing soluble inflammatory molecules), thus contributing to the chronic dysregulated AD immune response and exacerbating neuroinflammatory cycles [11]. This is a key point when taking into account the self-amplificating character of neuroinflammation.

Besides the release of cytokines and chemokines, one of the most effective signals for the detection and containment of pathogenic stimuli is the recognition of Damage-Associated Molecular Patterns (DAMP) [20]. DAMPs are a heterogeneous group of molecules that act as activation signals for early innate immune response in the presence of endogenous damaged tissues or external pathogens. This large group of molecules includes both Pathogen-Associated Molecular Patterns (PAMPs), which are compounds relased by exyternal pathogens, and “alarmins”, which are endogenous, paracrine factors secreted by the damaged cells (i.e., small compounds, metabolites, and/or cellular debris) [20].

Studies on Alzheimer’s diseased human brains and in transgenic AD models have demonstrated that amyloid-b peptides and neurofibrillary tangles can act as alarmins, by triggering the pattern recognition receptors (PRRs) system in both glial cells and neurons [21]. Regarding the role of external pathogenes, it has been demonstrated that pathogenic microbes may contribute to neurodegeneration in AD and that gut microbiota could contribute to driving an immunological response, leading to AD pathophysiology [22]. Among the alarmins, the proteins belonging to the high-mobility group superfamily (HMG), more precisely the high-mobility group box protein 1 (HMGB1), play a key role in driving the neuroinflammation leading to AD. HMGB1 can be passively released by necrotic cells or actively secreted from a variety of cells in response to dangerous stimuli. HMGB1 can trigger and amplify inflammatory responses [23]. HMGB1 has also been proven to generate and perpetuate neuroinflammation in AD by promoting both central and peripheral innate immune responses, thus playing a crucial role in the early AD pathogenetic cascade [24,25].

Glial cells are extremely sensitive to this class of compound. Indeed, these cells play a key role in monitoring the central nervous system microenvironment for signs of potential sources of tissue damage, including the presence of cancer cells. This is an interesting aspect when considering that glial cells have extremely aggressive oncogenic capabilities (as proven by the dramatically malignant nature of glioblastomas). Notably, despite the agreement on the existence of an inverse association between AD and neoplastic diseases (patients with AD have a low risk of developing cancer and vice-versa) [26], glioblastoma is an exception. Indeed a direct co-morbidity relationship has been shown between AD and glioblastoma [27,28].

There is a link between these two apparently opposite pathological conditions (glioblastoma is characterized by increased cell growth, AD by increased cell loss) in the dysregulation of the response to inflammatory stimuli. Again, a crucial role seems to be played by high-mobility group proteins (HMG), in particular the group A1 (HMGA1), since its expression has been found to be increased both in glioblastoma [29] and AD [30].

A number of studies have connected HMGA1 to the AD pathogenesis [30,31,32]. In particular, it has been demonstrated that HMGA1 interferes with the normal splicing of presenilin 2 (which constitutes the gamma-secretase, a critical enzyme for the production of amyloid β). In vitro studies have shown that HMGA1 binds to a site within exon 5 and inactivates normal splicing, leading to the generation of a truncated presenilin 2 protein [33]. The increased levels of HMGA1 proteins in patients with sporadic AD is likely due to hypoxia in neuronal cells [30,31,32,34,35]. Hypoxia would seem to favour the accumulation of amyloid β, as well as impairing tau phosphorylation and contributing to the degeneration of neurons and promoting the innate immune system [36], thus perpetuating the vicious circle that promotes the pathogenesis of AD. 

Another compelling analogy between neoplastic and AD pathophysiologies is the presence of cells in active cell cycles in both conditions. A high degree of active cell cycle is the typical condition of neoplasm, and interestingly, has been found in AD patients [37]. It has been demonstrated that the exposure of hippocampal adult neuronal cells to fibrillar beta amyloid is capable of inducing a cell cycle [38]. However, the neuronal cell cycle was abortive and a broad cellular stress with neurodegeneration was the result.

The role of the PI3K/Akt/mTOR (phosphoinositide 3 kinase/Akt/ mammalian target of rapamycin) pathway in cell cycle promoting has been proposed as a common intracellular signal transductor between AD and neoplasms. In addition, it has been hypothesized that cell proliferation and survival dysregulation may favour neurodegeneration in AD [26]. The PI3K/Akt/mTOR axis has been proposed to be crucial also in regulating defence mechanisms in the innate immune system [39]. In this scenario, this pathway may be able to sufficiently modulate immune activation in AD patients to constitute a promising therapeutic target [40].

Finally, recent studies suggest that innate immunity plays a pivotal role in regulating adult neurogenesis. Neurogenesis is a very important issue when considering AD pathophisiology. Adult hippocampal neurogenesis is crucial in learning procedures, and both verbal [41] and spatial [42] memory, which are usually impaired in AD. In murin models of AD, impaired neurogenesis plays a key role in pathological cascade, leading to cognitive decline [43]. Importantly, the defective adult neurogenesis appears to be driven by neuroinflammatory phenomena. A similar effect was recently reported in human subjects with AD [44].

**Figure 2:**
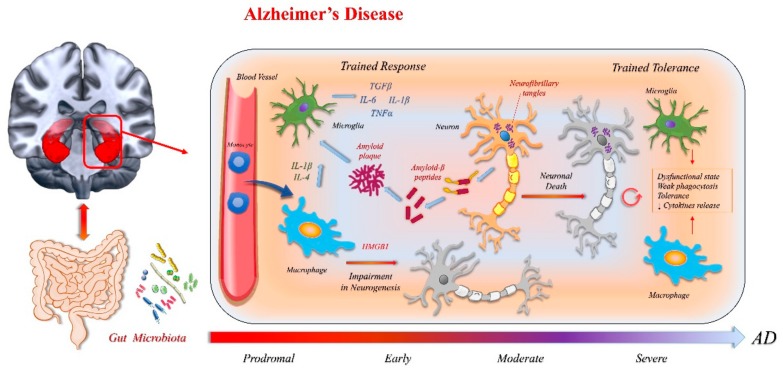
Immunological mechanisms associated with the pathogenesis of Alzheimer’s disease.

Impairment in neurogenesis has been found right from the early stages of the disease, even before the diffuse deposition of neurofibrillary tangles or senile plaques [44]. It is assumed that these anomalies also occur during the prodromal stages of the disease, which begins many years before symptoms appear [45]. The neurogenetic processes strictly depend on both innate and adaptative immunity. In response to inflammatory stimuli driven by microglia, macrophages, and lymphocytes, the multipotent neural stem and progenitor cells direct their cellular differentiation. Although it is not clear exactly how the immune response influences neurogenesis, pathways activated by Toll-like receptors (TLRs) are involved [46] and soluble inflammatory factors are crucial. Among these, proinflammatory cytokines (IL-1β, TNFα, IL-6) are able to stop neurogenesis, whereas anti-inflammatory cytokines (IL-4, IL-15) and trophic factors (IGF-1, BDNF) seem to promote it. Even more importantly, the effects of these soluble mediators change according to the microenvironment in which they operate [47]. Finally, intestinal microbiota regulate neurogenesis through an immuno-mediated response [22], and the cross-talk between immune cells and local neural progenitor cells is thus a complex and multifaceted phenomenon in which local and peripheral tissue factors are crucial. Figure 2 summarizes the main mechanisms involved in the pathophysiology of AD.

### 2.2. Parkinson’s Disease

Parkinson’s disease (PD) is a progressive degenerative disorder of the CNS, which mainly affects the motor system. The deposition of misfolded protein aggregates (mainly composed of α-synuclein) in the substantia nigra pars compacta and the progressive degeneration of dopaminergic neurons are classically considered the neuropathological hallmarks of PD [48,49]. The main clinical symptoms of the disease (tremor, rigidity, and bradykinesia) reflect the underlying basal ganglia neurodegeneration and protein deposition. This classical view has now been broadened to other non-motor related brain regions. Ideed, there is evidence that other areas may be affected by neurodegenerative phenomena before the involvement of the substantia nigra (autonomic and enteric nervous system, olfactory bulb, medulla oblongata, and pontine tegmentum), or later over the course of the disease (neo-cortex). This produces a much more complex non-motor symptomatology, including olfactory loss, sleep disturbance, depression, autonomic dysfunction, and cognitive impairment [50,51].

There are still many open issues regarding the mechanism that triggers neuronal cell death and the misfolded protein accumulation in PD. It has been postulated that alterations in the physiological immune response play a key role in the pathogenesis of PD. Several converging findings deriving from different study modalities underline the important role of microglia in PD development:

(i) Post-mortem pathological studies. Examinations of histopathological samples from human Parkinson’s diseased brains have revealed a wide distribution of activated microglia (positive for the MHC class II) in several brain regions, including the striatum, often in combination with α-synuclein-positive Lewy neurites [52,53,54]. In addition, inflammatory mediators which are released by, or which promote the activation of, microglia have been identified in the brain tissue of PD patients. Indeed, a high level of the CXC-family chemokine ligand 12 (CXCL12) and its receptor CXC-family chemokine receptor 4 (CXCR4) [55], as well as TNFα, IL-1β, interferon γ (IFNγ), nitric oxide synthase (NOS), and reactive oxygen species (ROS) have been found in the nigral tissue. Higher concentrations of the proinflammatory interleukins (IL-1β, IL-2, IL-6) and TNFα have also been found in the striatum [56,57,58,59].
(ii) In vivo studies on biological samples (cerebrospinal fluid and blood). The same panel of proinflammatory mediators have been found both in cerebrospinal fluid (IL-1β, IL-6, and TNFα) and blood (serum and plasma) samples [58,59,60,61,62,63,64], thus confirming the role of these microglial-related inflammatory mediators.
(iii) In vivo PET imaging studies. Studies that used the PK11195 PET ligand, a selective ligand for the peripheral benzodiazepine binding site (PBBS), which is considered a selective marker of in vivo microglial activation, demonstrated a widespread microglial activation in early stages of PD disease [65], but not in the late course of the disease. The new highly specific DPA714 (N,N-diethyl-2-[4-(2-fluoroethoxy)phenyl]-5,7-dimethylpyrazolo[1,5-a]-pyrimidine-3-acetamide) PET ligand has also been used to measure the regional distribution of activated microglia in PD patients, showing neuroinflammation within the substantia nigra of the most affected hemisphere [66]. In addition, the P2X7 (P2X purinoceptor 7 receptor) PET ligand revealed microglial P2X7 availability in acute but not chronic rodent models of PD [67]. Taken together, all these findings demonstrate the key role of activated microglia in the regions critically involved in PD, which takes place early in PD development.

But what stimulus triggers the activation of microglia? Several studies conducted in vitro and on animal models for PD have addressed this question. Although this is currently a matter of debate, the abnormal deposition of misfolded α-synuclein is one of the major suspected triggers. Two different approaches—transgenic mouse models, and recombinant adeno-associated viral vector-based α-synuclein rodent and primate models—have revealed that α-synuclein can initiate neuroinflammation, before the neurodegeneration has occurred [68,69,70]. Other candidates are the matrix metalloproteinase 3 (MMP3, a proteinase that cleaves the extracellular matrix) and neuromelanin (a dark pigment, consisting of a tangled aggregate of melanin, peptides, and lipidic components), which are released into the extracellular space upon dopaminergic neuron damage [71,72]. In particular, neuromelanin binds toxic metals and catecholaminergic products, inducing oxidative stress and local microglial activation, thus perpetuating the cycle of reactive microgliosis in Parkinson’s disease [73,74,75]. 

Whatever the trigger for microglial activation, the microenvironment plays an important role in activating and maintaining the inflammatory process. Notably, the microenvironment is involved as a source of elements that are both directly and indirectly detrimental to the dopaminergic neurons, through the activation of microglia. Interestingly, the effects of recurrent external environmental insults have been considered critical for microglial activation in PD, and the “multiple hit” hypothesis has been postulated, according to which multiple environmental exposures could drive the development of the disease [76]. Several environmental elements have been found to be involved in PD pathogenesis, including infectious agents [77,78], pesticides [79], 1-methyl-4-phenyl-1,2,3,6-tetrahydropyridine (MPTP) [80], and heavy metals [81,82,83]. 

The gut microbiota has attracted much interest in recent years. It regulates the development and function of microglia and astrocytes, modulating peripheral immune responses, with important consequences for brain inflammation, and the gut microbiota–immune axis likely plays a key role in PD development [22].

Astrocytes would also seem to play an important role in maintaining inflammatory phenomena in PD, together with microglia [84,85]. Astrocytes share many important functions with microglia in terms of the maintenance of neuronal trophic support, the control of synaptic homeostasis, the proinflammatory role, and phagocytic activity [86]. There is a strong interaction between microglia and astrocytes through several mediators reciprocally released from both cell types [87]. Inflammatory stimuli likely trigger reactive astrocytes, which can be classified into two different phenotypes with opposing functions: (i) A2 astrocytes, which have neuroprotective functions, [88]; and (ii) A1 astrocytes, which result in a neurotoxic action [88,89]. The A2 to A1 phenotypic conversion of astrocytes is actively promoted by reactive microglia through IL-1, TNF, and C1q factors [90]. Neurotoxic A1-activated astrocytes have been found in the post-mortem tissue of patients with PD, suggesting that they promote neurodegeneration [88,90]. In vitro studies have demonstrated that astrocytes are able to phagocytize fibrillar α-synuclein [91,92], however they tend to accumulate α-synuclein in intracellular agglomerates rather than degrade it. 

In agreement with the above findings, pathological studies from PD subjects have revealed the diffuse presence of astrocytes characterized by broad intracellular synuclein deposits in the brain regions critically involved in the disease [93,94,95,96,97]. Internalization without the cleaving of fibrillar oligomeric α-synuclein has a detrimental effect on the lysosomal and mitochondrial homeostasis of astrocytes, which react by actively transferring the synuclein aggregated to nearby astrocytes via direct contact and tunneling nanotubes, thus perpetuating neuroinflammation [91,92,98]. Ineffective cleaving of α-synuclein by activate astrocytes might therefore play a key role in PD pathogenesis, with particular effects on the disease propagation to nearby areas.

Interestingly, some of the most important genes linked to familial forms of PD are involved in taking up and degrading extracellular materials. These genes are highly expressed in microglia and astrocytes, given the substantial implication in endolysosomal and autophagic pathways. 

Among PD-associated proteins, ATPase type 13A2 (ATP13A2), glucocerebrosidase (GBA), and leucine-rich repeat kinase 2 (LRRK2) are specifically located at the lysosomal level and/or are crucial for lysosomal functionality. Mutations in the ATP13A2 gene cause autosomal recessive PD, mutations in LRRK2 gene cause autosomal dominant or sporadic PD [99,100], while homozygous GBA mutations represent frequent risk factors for PD and other synucleinopathies [101,102,103]. 

LRRK2 is highly expressed in mouse and human astrocytes and is key to the astrocytic lysosomal function [104,105,106], as well as in microglia and monocytes, more than in neurons, thus highlighting its crucial role in the innate immune system. LRRK2 intrinsically regulates microglial activation and autophagolysosomal degradation [107,108]. Indeed, in mouse primary astrocytes, LRRK2 co-localizes with the lysosomal markers, and mutations in LRRK2 generate larger lysosomes, but with a lower proteolytic power [105]. Therefore, lysosome alterations induced by LRRK2 malfunction in astrocytes might contribute to the neurodegenerative phenomena, inducing the inability to remove dying neurons and the release of α-synuclein agglomerates.

Recessive PD genes are also involved in the neuroinflammation underlying PD. 

The expression of PINK1, Parkin, and DJ-1 is higher in reactive astrocytes in the diseased human brain [109,110,111], suggesting that these proteins play a key role in modulating glia-dependent immune responses. The DJ-1 gene is an autosomal recessive gene linked to PD [112,113]. DJ-1 was initially identified as an oncogene and its expression was found to be enhanced in several types of cancers [114,115]. It is involved in the regulation of gene transcription and exerts an antioxidant activity; it could also be involved in mitophagy phenomena [116]. A loss of DJ-1 function in microglia cells reduces the expression of lipid raft on cellular surfaces and reduces their ability to internalize and degrade the a-synuclein, probably by influencing the autophagy ability [117]. The loss of DJ-1 has been found to lead to the highly increased expression of IL-6 and other proinflammatory mediators by lipopolysaccharide-treated astrocytes and may be related to the lower subsistence of co-cultured neurons, in comparison with non-treated astrocytes [118]. The PINK1 gene is responsible for a recessive PD [119,120], and it codes for a protein-kinase that regulates mitochondrial functions by promoting the mitophagy of depolarized mitochondria [121]. PINK1 deficiency is likely associated with increased levels of NO, heightened oxidative stress sensitivity, as well as abnormal mitochondrial function and morphology in glia cells [122].

**Figure 3:**
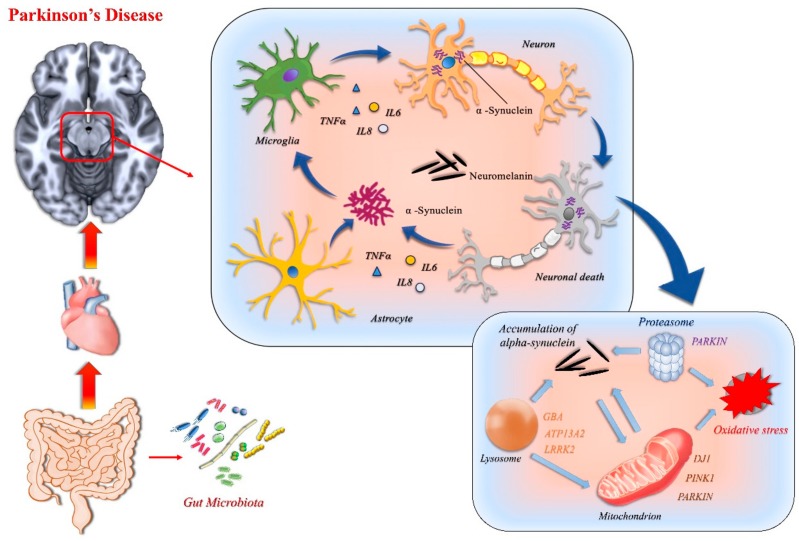
Biochemical and immunological mechanisms associated with the pathogenesis of Parkinson’s disease.

In conclusion, since mitochondrial metabolism and lysosomal degradation act as a key final step to resolve protein aggregation upon glial phagocytosis, the presence of mutations in genes that regulate this function adds further susceptibility to the development of neuroinflammatory phenomena involving innate immunity. Figure 3 summarizes the main processes involved in PD.

### 2.3. Huntington’s Disease

Huntington’s disease (HD), also called Huntington’s chorea, is a genetic, autosomal dominant disease, caused by the expansion of a trinucleotide sequence (CAG) in exon 1 of the huntingtin gene. The CAG repeat causes the synthesis of an aberrant protein, which adversely affects the brain. In the classical disease variant, involuntary movements, psychiatric, and cognitive dysfunctions occur, with an inexorably poor prognosis [123]. 

Exactly how the mutated huntingtin induces the degenerative phenomena underlying the clinical symptoms is not fully understood. Physiologically, the huntingtin seems to have multiple biological functions, including axonal and vesicular transport, endocytosis, post-synaptic signaling, and cell survival pathways [124]. The mutant huntingtin is prone to cleavage, which then creates shorter fragments containing the N-terminal polyglutamine expansion, which oligomerize and form aggregates that have been implicated in neurotoxicity [124]. The dysregulation of several functions occurs, including gene transcription, axonal transport of critical factors, calcium signaling, protein interactions, autophagy, and proteasomal and mitochondrial alterations [125]. Despite the fact that the huntingtin gene is expressed ubiquitously, the striatum and cortical areas are the most affected regions. 

Although less explored than in other chronic degenerative diseases, the role of the immune reaction and neuroinflammation have been highlighted. Mutated huntingtin may promote the inflammatory response by a direct toxic effect, or indirectly, via mitochondrial dysfunction, as the huntingtin gene is also highly expressed in microglia and in peripheral immune system cells [126,127,128,129,130]. Innate immunity seems to play a more important role than adaptive immunity in HD. Indeed, the accompanying infiltration of adaptive immune cells into the central nervous system has only rarely been found in HD [131,132]. 

Several studies suggest that activation of the immune response in HD occurs even in the preclinical stages of the disease [133]. Histopathological examination of the brains of mutant huntingtin carriers revealed the presence of activated microglia before the onset of symptoms [134]. In addition, microglial activation was found to be related to both disease progression and the depletion of terminals binding the dopamine D2 receptor [135,136]. 

PET studies have used the PK-11195 ligand to mark activated microglia [137], and high levels of microglial-related proinflammatory cytokines were found in the plasma of HD patients and in murine models of the disease [133], further confirming the critical role of microglia in HD. Thus, the neuroinflammation likely occurs early in the pathogenetic cascade of events and triggers striatal and cortical neurodegeneration.

In addition to microglia, astrocytes appear in the affected regions. Moreover, given the ubiquitous expression of the huntingtin, changes in the immunologic cells are probably not limited to those resident in the central nervous system, but may involve the peripheral immune cell populations. The migratory capabilities of macrophages have been widely studied. An impaired migration of macrophages in response to chemotactic stimuli has been found in mouse models of HD. Notably, this impairment was governed by the mutant huntingtin expression [138]. Migration defects have also been found in human monocytes and macrophages of HD patients [138]. 

**Figure 4:**
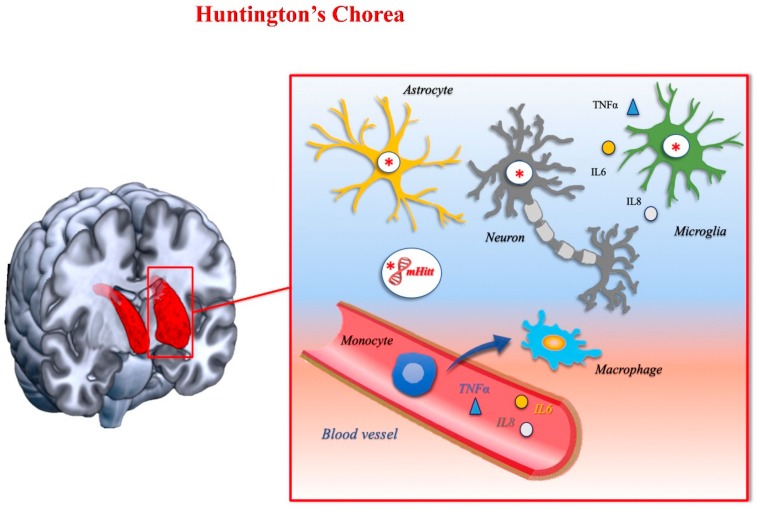
The immunological mechanisms associated with Huntington’s disease. *mHtt: mutated huntingtin.

Based on this evidence, a dysfunction in the chemoattractant mechanisms has been proposed in HD. A defective migration may adversely impact the release of cytokines and chemokines, producing a chronic increase in the levels of proinflammatory cytokines and chemokines in the central nervous system. In turn, this may trigger microglial activation with a cascade of proinflammatory phenomena, which then lead to neurodegeneration [138]. Figure 4 shows the main immune mechanisms involved in HD.

### 2.4. Amyotrophic Lateral Sclerosis and Frontotemporal Dementia

Amyotrophic lateral sclerosis (ALS), also known as motor neuron disease (MND), is a progressive degenerative disorder affecting both the upper and lower motor neurons. This gradually leads to stiff muscles, muscle twitching, and weakness, with loss of voluntary movement [139]. Cognitive and/or behavioral dysfunctions may also be present, thus constituting the non-motor manifestations of ALS, characterized by personality alteration, irascibility, fixed ideas, poor insight, and prevalent impairment in frontal functions. This clinical picture is congruent with the changes in temperament, social behavior, and executive function occurring in frontotemporal dementia (FTD) [140]. 

ALS and FTD are neurodegenerative diseases with clear similarities in terms of clinical, genetic, and pathological findings. Based on clinical criteria, these two conditions can co-occur in the same patients. Indeed, up to 50% of ALS patients may have FTD features, while about 15% of patients with FTD show a motor neuron disease [141,142]. Both are chronic neurodegenerative disorders, with a poor prognosis, and there is currently no pharmacological treatment to curb the fatal evolution of the disease. 

Neuroimaging, neuropathological data, and genetic studies suggest that ALS and FTD might form part of a disease continuum [143,144,145]. Indeed, a number of genes linked with ALS and FTD are involved in the same cellular pathways. The expression of many of these genes is not limited to neurons, but also involves glial cells, suggesting a multicellular pathogenesis, including those of innate immunity. 

Although the cause of ALS and FTD is still unknown, the impact of the innate immune response has been suggested as being important for both the initiation and the progression of these diseases [146,147,148]. 

Neuroinflammation and microgliosis are broadly demonstrated in ALS. In post-mortem affected tissues, the upregulation of factors related to the innate immune response (i.e., molecules of complement activation pathway, chemokines, MHC class I and II, and integrins) has been reported [149]. Pathological studies in ALS patients also show the presence of dendritic cells and activated microglia/macrophages in the brain [150], as well as increases in microgliosis and astrogliosis, especially in the spinal cord [151,152,153,154]. Evidence has also been found of early innate immune response in the motor cortex of ALS patients and mice models of the disease [155]. 

Innate immune-mediated mechanisms also play a key role in FTD. In vivo PET imaging with the PK11195 ligand, which identifies inflammatory glia, has revealed the presence of activated microglia in the frontotemporal lobes of patients with FTD, and this finding was evident before the atrophy occurred [156]. Pathological studies in human FTD cases have revealed ubiquitin-positive and TDP-43 (transactive response DNA-binding protein 43KDa) inclusions localized predominantly in the frontotemporal cortex, but also in the brain stem and spinal cord, involving both gray and white matter. Of note, based on morphology and double-labeling experiments, the white matter cells with TDP-43-positive inclusions were oligodendrocytes and other glial cells, thus demonstrating that glial pathology contributes significantly to the neurodegenerative processes involved in FTD [157].

Taken together, all these data highlight the crucial role of innate immune responses both in ALS and FTD, whose activation is initially aimed at restoring tissue homeostasis, but ultimately generate a vicious circle by promoting proinflammatory toxic phenomena [147,158,159,160]. 

Neuroinflammation triggers a perturbed balance in bioenergetics functions and a large increase in the production of ROS [161]. Oxidative stress is considered to be crucial in the pathogenesis of ALS [162]. This is widely demonstrated in animal models of motor-neuron disease and in ALS patients. Indeed, mutations in the superoxide-dismutase (SOD1) gene are responsible for most of the familial forms of ALS [163]. The SOD1 gene is highly expressed in microglia and astrocytes, and several experiments to evaluate the effects of selective expression of the SOD1 mutations in different cellular lines have shown that the non-neuronal cells surrounding the motor neurons, and in particular microglia, are critical for ALS progression [164]. 

Cultured microglia expressing mutant human SOD1 can promote motor neuron death [165]. Moreover, decreased SOD1 activity promotes the accumulation of superoxide radicals with toxic activity [166]. Therefore, together with the increase in oxidant production (as a result of inflammatory phenomena), there is a reduction in antioxidant response, with a detrimental spiral of effects that ultimately leads to the amplification of toxic phenomena and to degeneration. 

The hypothesis that altered microglial activity plays a substantial role in the pathophysiology of both ALS and FTD is strongly supported by evidence that ORF 72 on chromosome 9 (C9orf72), the gene most frequently linked to ALS/FTD [167], has a high impact in myeloid cells. C9Orf72 encodes a protein that is active in the regulation of endosomal trafficking and which interacts with proteins involved in autophagy and lysosomal activity. C9Orf72 is highly expressed in myeloid cells, and the loss of function of this protein produces a pro-inflammatory state, with high levels of pro-inflammatory cytokines, lysosomal accumulation, and hyper-reactive immune responses, thus altering myeloid cell function and immunity [168]. 

On the other hand, the most important features of C9Orf72 in ALS/FTD patients is the neuronal accumulation of intracytoplasmic aggregates containing dipeptide repeat (DPRs) proteins [169,170,171,172]. These endogenous DPR proteins appear to be highly toxic, producing altered RNA metabolism, and the disruption of nucleocytoplasmic transport [173,174]. Therefore, C9orf72 likely has a different effect on different cell types: proinflammatory in microglia, and protein aggregate toxicity in neurons. These combined effects may lead to neurodegeneration in ALS/FTD [147,168,175].

Microglia might not be the only glial population that contributes to neuroinflammation. There appears to be a complex interaction between microglia, astrocytes, and neurons in FTD. Astrogliosis, occurring since the early stage of the disease, has been found in FTD [176,177], and the expression of apoptotic markers (such as caspase-3) and morphological changes were found in astrocytes [176]. Astrocytic action is also crucial in ALS pathogenetic phenomena, which are modeled as a complex multifactorial cascade of events in which innate immunity has a pivotal role in regulating the speed of disease progression [178].

Several studies in both human cases and experimental models of ALS suggest that a critical factor for ALS progression is the astrocytic action. Astrocytes in ALS are active players in neuronal injury by producing neurotoxic mediators [179,180,181,182,183]. They also indirectly cause neuronal sufferance because they are themselves subject to death, thus depriving the motor neurons of their supportive action, thus accelerating neuronal cell death [184,185,186]. Several findings suggest that anomalous activation of the complement system, not only in the central nervous system but also peripherally, may be implicated in the pathophysiological phenomena of ALS [164]. Deposition of C3d and C4d near to degenerating motor neurons in proximity to CR4+ microglia has been demonstrated [153], as well as high levels of C1q and C4 during the disease progression in SOD1 mice [187]. Post-mortem studies in human ALS patients further confirmed this evidence, showing an increased production of C1q and C4 complement factors by neurons and microglia [188]. Moreover, increased C3 and/or C4 levels were found in the cerebrospinal fluid of ALS patients [189,190,191]. The increased levels of C1qB, C4, C4d, and factor B were found to be related to the disease progression, thus suggesting that the complement system is a critical effector of the destruction of motor neurons during the course of the disease.

The complement deposition (C1q, C3b/iC3b, and C5b-9 factors) in proximity of the neuromuscular junction, motor neuron terminals, and near the Schwann cells occurs early, also in presymptomatic stages, both in human ALS patients and in transgenic SOD1 mice [192,193], leading to a peripheral axonopathy that precedes the central neuronal death. This evidence supports the “dying-back” hypothesis, which has raised much attention in the context of ALS pathophysiology. According to this hypothesis, ALS may start as a distal axonopathy, so the alterations take place first distally at the neuromuscular junction level and then move proximally towards the cellular body [193,194,195].

**Figure 5:**
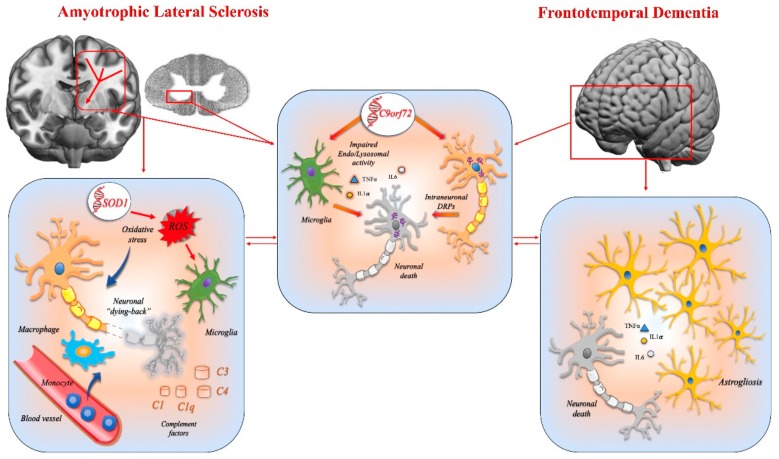
The immunological mechanisms associated with amyotrophic lateral sclerosis and frontotemporal dementia.

In this context, the complement system could play an important role: (i) by activating a peripheral innate immune response (i.e., monocytes) at the neuromuscular junction level; and (ii) by creating an immune-activating environment with microglia in the central nervous system, thus extending the degeneration during the progression of ALS. Further evidence supporting backward degeneration involving first the neuronal axons derives from the demonstration that in the central nervous system degenerative changes occur in oligodendrocytes, a cellular population that is crucial for axon support. A significant loss of oligodendrocytes in both ALS patients and presymptomatic ALS mice has been described [196,197], and in the surviving oligodendrocytes, a decreased expression of monocarboxylate transporter 1 (which is essential for oligodendrocytic trophic function) has been found. Oligodendrocytes may therefore also have a role in ALS-related motor neuron axonal degeneration. Figure 5 shows the main mechanisms involved in ALS and FTD.

## 3. Psychiatric Diseases

Immune dysregulation plays a role not only in neurological autoimmune brain diseases, such as multiple sclerosis, and in neurodegenerative illness, but also in many psychiatric disorders. Microglia, which represent the population of brain-residing phagocytes, can be activated to carry out the complex functions of innate immunity, in the context of inflammatory or infectious events. However, they can also interface with neuronal cells during embryonic and adult life, regulating their activity and therefore the quantity and quality of synaptic connections. These events represent the basis of subtle morpho-functional brain alterations that could influence the development of psychiatric diseases. Microglia also contribute to physiological developmental processes in CNS: they not only control the local environment by their specific processes, but during brain development, they play an important role in synaptic pruning, i.e., phagocytizing the complement-tagged synapses with the mediation of C1q and C3 complement proteins. In the adult brain, microglia play a key role in the homeostasis of synaptic circuits. The expression of purine receptors and for excitotoxic neurotransmitters enables them to sense local neuronal activity. Microglia can directly respond to diverse stimuli by physically contacting neurons via outgrowing processes and also indirectly, by modulating the neuronal firing rate by signaling molecules such as the tumor necrosis factor or by releasing extracellular vesicles [198].

Innate and adaptive immune mechanisms work in infection and autoimmunity. Infections can trigger chronic inflammation, which damages cognitive function. In humans, it has been observed that prior hospitalization for autoimmune disease or infectious disease can increase the risk of a major mood disorder by 45% and 62%, respectively. Studies with rodents have revealed that infections and systemic inflammation in the fetus, during the prenatal or perinatal period, can cause long-term cognitive damage. Such models could explain why early infections increase the risk of young adulthood psychosis [198].

### 3.1. Depression 

Depression is a widespread chronic illness that can affect thoughts, mood, and physical health. It is characterized by sadness, low mood, lack of energy, insomnia, and anhedonia. A link between depression and increased inflammatory response was reported by Maes and colleagues and led to the formulation of the so-called “cytokine hypothesis” of depression, which is also known as the “macrophage hypothesis” [199,200,201,202]. According to Maes and coworkers, increased interleukin production, such as Il-1β and Il-6, by monocytes in severe depression might underlie the various aspects of the immune and “acute” phase responses detectable in major depressive disorders. 

In addition to the “macrophage hypothesis”, there is now also the “mast cell hypothesis”. Mast cells (MCs) are long-lived cells, associated with chronic inflammation and allergic reactions, and which contain cytoplasmic granules filled with pro-inflammatory substances. Tissue injuries can cause MC degranulation and the release of cytokines and other molecules that can trigger the inflammatory response. MCs are involved in many pathological processes in the CNS or elsewhere, both by secreting pro-inflammatory cytokines and by the action of enzymatic factors [4]. 

IL-1, a multipotent cytokine, activates MCs and forces them to release several inflammatory compounds and chemokines, including IL-6. In fact, IL-6 can sustain inflammation even without degranulation and tryptase release by normal MCs. 

Depression may lead to MC activation and inflammation, with increased secretion of IL-1 and inhibition of IL-37. Many recent studies and meta-analyses have reported that levels of inflammation markers, such as TNFα, IL-6, and C-reactive protein (CRP), are high in depressed individuals, thus indicating a dysregulation in the immune system [203,204]. Mood changes in humans, subsequent to the injection of the typhoid vaccine [205] or lipopolysaccharide (LPS) [206], have been shown to accompany the hyper-production of pro-inflammatory compounds that modulate the toll-like receptors (TLRs) pathways [207]. Interestingly, endotoxin administration in healthy subjects compared to placebo increased serum levels of TNF and IL-6, and caused mild depressive-like symptoms, whereas sub chronic pre-treatment with the serotonin-reuptake inhibitor citalopram blunted the endotoxin-induced mood symptoms [208]. 

About 20–50% of hepatitis C and cancer patients who have undergone interferon-α (IFNα) therapy are estimated to develop clinically significant depression [209], associated with high serum levels of sIL-2r, TNFα, and IL-6 [210]. Likewise, with regard to LPS-induced depression, for IFN-α there is evidence of a positive response to conventional antidepressant treatments. This positive response is consistent with the hypothesis of shared pathways between inflammation and idiopathic major depression [211,212,213]. The chapter on “Depressive Disorders” in the *Diagnostic and Statistical Manual of Mental Disorders* (5th ed., American Psychiatric Society) suggests that this disease may be generated directly from other pathologies already present in the patient. Some of these conditions can trigger mechanisms that involve components of the innate immunity system [214]. Primitive brain tumors may have a relationship with psychiatric symptoms, such as depression, due to lesion activity and mass effect. Classically, incidence differs in relation to the size of the neoplasia [215]. However, the most important neoplasms, such as glioblastomas, are immunologically “cold” tumors due to their tendency to suppress the immune response.

Interestingly, CNS neoplasms with high immunogenicity, such as the choroid meningioma that can evoke a Castleman-like syndrome, could be associated with depressive symptoms, probably due to innate immunity imbalances [216,217,218]. A confirmation of the role of inflammation in generating depression may be further found in Castleman’s disease, in its classical form [219], and in other diverse neoplasms that can trigger the same mechanisms, such as cardiac myxomas [220,221,222]. The main link between such inflammatory pathologies and depressive symptomatology could be the IL-6 secretion by activated macrophagic cells and, in turn, IL-6 modulates almost every aspect of the innate immune system [223].

In other cases, mood disorders and behavioral dysfunction in association or before the clinical evidence of neurological symptoms have been reported in paraneoplastic syndromes. Lung cancer is the leading cause of cancer-related mortality worldwide. Moreover, pulmonary cancers and other types of neoplasms can trigger complex autoimmune mechanisms, coupled with the production of autoantibodies that sustain limbic encephalitis (LE) [224,225,226]. Psychiatric changes, such as irritability, depression, hallucinations, personality disturbances, and cognitive changes, are commonly described in LE. Factors belonging to innate immunity may also be involved in the LE pathogenesis [227]. 

The body mass index (BMI) also plays an important role in the association between depression and inflammation through inflammatory cytokine levels [228]. Chronic over-nutrition and obesity induce a chronic low-grade inflammation state throughout the body, called “metainflammation”. This state is accompanied by a higher number of M1 polarized pro-inflammatory macrophages, found within the colon, liver, muscle, and adipose tissue [229]. Via this mechanism, innate immunity probably exerts a pivotal role in bridging obesity and depression. The high degree of comorbidity between depression and anxiety disorders [230] could be at least partially due to an overlapping etiology. A dysregulation of the immune system also occurs in anxiety disorders and it has been associated with higher hematic levels of CRP [231,232]. 

Bipolar disorder has its own immunological fingerprint [233], which is discernable different from that of schizophrenia [234,235] and panic disorders. There may be an etiological connection between bipolar and panic disorders and mannan-binding lectin (MBL) deficiency, a component of the lectin pathway of complement activation [236]. 

Finally, the role of the brain–gut axis in depressive disorders is receiving increasing attention. Depression and generalized anxiety disorder can be associated with gastrointestinal disturbances. Epidemiological studies have shown that over 50% of patients with irritable bowel syndrome (IBS) show comorbidity with sleep troubles, depression, or anxiety. There is a bidirectional connection between the gut microbiome and sleep and depression through neuroendocrine, immunoregulatory, and autonomic pathways. Neuroinflammation can be involved in these circuits because changes in intestinal permeability facilitate the recognition of bacterial lipopolysaccharide by toll-like receptors on the surfaces of the immune cells of the intestinal mucosa. This elicits the secretion of pro-inflammatory cytokines, which in turn causes an inflammatory response in the brain [237,238]. Interestingly, alterations in the physiology of the brain–gut axis are also present in PD and AD. Such types of changes may also play a role in the genesis of psychotic troubles (see below). 

Neuropsychiatric syndromes are common in neurodegenerative disorders. They occur in the majority of patients with AD. Depression and psychosis are also associated with more rapid cognitive decline in AD [239]. Although the classic doctrine suggested that depression in early life may be a risk factor for a subsequent dementia diagnosis and that depression in late life is a prodrome of dementia, the results of recent studies indicate higher odds for developing dementia after depression, even when the depression occurred 20 years or more before the dementia [240]. Neuroinflammation via low molecular weight, soluble proteins, or produced by activated microglia may represent a major link between depression and AD. In fact, the patterns of circulating pro-inflammatory cytokines that increase in depressive disorders are the same in AD [241].

**Figure 6:**
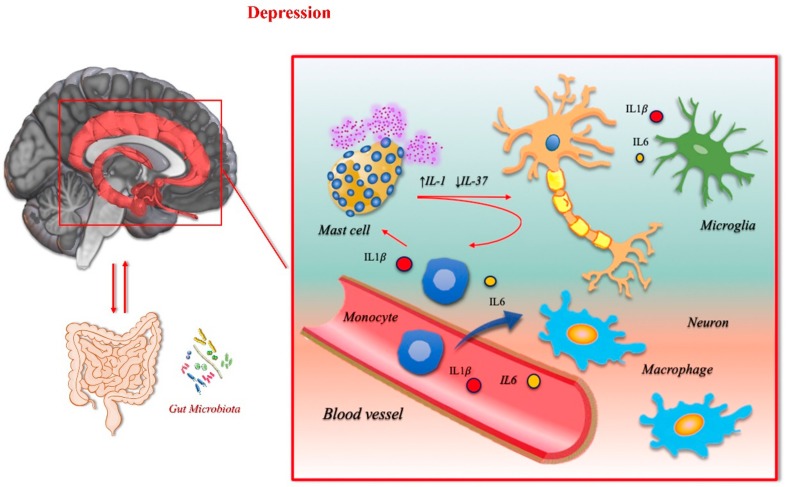
The immunological mechanisms associated with depression.

The role of inflammation in the genesis of the depression is further supported by the microglial activation and neuroinflammation found in the brains of patients with depression examined post-mortem. Increases in activated microglia or perivascular macrophages in suicide victims have been reported in the parenchyma. In AD, microglia are also activated and abut the core of amyloid plaques [242]. These results suggest that non-steroidal anti-inflammatory drugs could perhaps be used to treat major depressive disorders. Interestingly, the increase in IL-6 in childhood enhances the risk of developing depression later in life [243], and there is a relationship between high IL-6 levels and depression in patients with PD [244]. Figure 6 reports the main mechanisms involved in depression.

### 3.2. Schizophrenia 

Schizophrenia is a highly disabling disorder whose causes are not completely understood. Although diagnosis has become more reliable with operational criteria, included in *Diagnostic and Statistical Manual of Mental Disorders, (DSM) Fifth Edition* [245], the validity of the disease boundaries remains unclear because of substantial overlaps with other psychotic disorders. 

Schizophrenia has a major genetic component, with about 80% of the variation in the trait of schizophrenia attributable to genetic factors. The genetic risk for schizophrenia is based on many common genetic variants, each with a small effect and a few uncommon genetic alterations with a larger impact on phenotype. Genes that confer risk for schizophrenia may also be associated with other psychiatric disorders [246]. 

Schizophrenia and bipolar disorder may arise from shared genetic factors, but the resulting clinical phenotype is influenced by additional alterations mediated by microglia, possibly caused by the interference of environmental factors at different times during neurodevelopment and early life or interactions between groups of genes and environment [234]. 

A growing body of evidence also suggests that schizophrenia and immune diseases share genetic risk factors. Genome-wide association (GWA) studies have shown a genetic overlap between schizophrenia and Crohn’s disease (CD), primary biliary cirrhosis, psoriasis, systemic lupus erythematosus, and ulcerative colitis (UC) [247]. Interestingly, CD and UC are inflammatory bowel diseases (IBDs), a group of chronic inflammatory disorders with multifactorial aetiologies that affect the gastrointestinal tract and extraintestinal organs. These disorders have another component in the perturbed crosstalk between the innate immune system and microbiota. They integrate all aspects of mucosal immunology at the interface between microbial colonization and innate immune system activation. 

Dysbiosis has a central role in the pathogenesis of IBD, but immune dysfunction is necessary for the development of this condition. Indeed, multiple mechanisms at the interface between the innate immune system and the microbiome likely contribute to the molecular pathophysiology of IBD. Genome-wide association studies of such diseases have found allelic variance in several genes that regulate the innate immune system [248]. Intestinal mucosa is a classic barrier belonging to the innate immunity system, but immune signaling is an important mechanism by which the microbiota–gut–brain interaction can operate: the cytokine production can be influenced by the gut microbiota, which can then signal to the brain via the circulatory system.

In schizophrenia, abnormalities such as increased activity and density of microglia and of pro-inflammatory serum cytokines have been found in many studies, including meta-analyses. Higher serum levels of IL-6, TNFα, and IL-1β have been recorded in first-episode drug-naïve patients and in chronic patients [249]. Both IL-12, required for Th1 cell differentiation and able to induce the increased production of IFNγ by macrophages, and TGFβ, with immunosuppressive and anti-inflammatory activities, are increased in the serum of schizophrenic patients [249]. These data confirm the importance of neuroinflammation in the genesis and evolution of the psychosis and that a complex cytokine trafficking underlies it.

Regarding schizophrenia, alterations in gut microbial profiles have been observed in preclinical models. Fecal microbiota transplant from schizophrenic patients into germ-free mice causes a schizophrenic phenotype in the animals. The presence of *Candida albicans* may perhaps also be associated with worse psychiatric symptoms in males with schizophrenia. Finally, there appears to be an association between schizophrenia severity and the specific genera of bacteria, including *Veillonella* and *Lachnospira* [250]. Imaging studies in schizophrenia have revealed reduced gray matter thickness and abnormal functional connectivity. Other postmortem studies reported a lower number of dendritic spines. 

Given the extensive elimination of synapses in the human cerebral cortex during late adolescence and early adulthood, excessive synaptic pruning by microglia may reduce synapse density in schizophrenic patients. Studies in rodents and in vitro using human-induced pluripotent stem cells (iPSCs) have confirmed that excessive engulfment and pruning of synapses, via the combined action of complement and microglia, may promote the development of the disease [251]. 

In synaptosomes isolated from cell cultures of patients suffering from schizophrenia, an increased amount of C3 complement was detected, which strongly correlates with the long form of the *C4A* gene (*C4AL)* copy number. In addition, the antibiotic drug minocycline, which may inhibit synapse engulfment by microglia, seems to protect against excessive synapse pruning, which is a key event in schizophrenia [252]. 

Another important role in psychiatric disorders is carried out by alterations in the BBB’s alterations [253]. This mechanism has been pointed out in schizophrenia and related psychoses [254,255,256,257]. Loss of BBB integrity precedes the rise of NADPH oxidase 2 levels (alias cytochrome b-245, encoded by *CYBA* and *CYBB* genes) in the prefrontal cortex of an animal model. Thus, BBB damage may be considered an epistatic event in brain alterations in this model [258]. Although all CNS cell types and their neurovascular units probably express one or more NADPH oxidase isoforms, microglia express by far the highest levels of NOXs, in particular NOX2. This is the classical phagocyte NADPH oxidase, and therefore such cells probably have a crucial role in BBB leakage in psychiatric illness [259]. Microglia NOX-derived oxidants also facilitate the paracrine modification of synaptic function through long-term depression and in communication with the adaptive immune system [260]. 

Psychotic symptoms characteristic of schizophrenia, such as hallucinations and delusions, can be associated with neurodegenerative diseases and require treatment [239]. Neuropsychiatric symptoms, such as apathy, social withdrawal, disinhibition, agitation, and psychosis, are common in the mid-to-late stages of AD. Proinflammatory cytokines play an important role in the pathogenesis of psychosis in AD patients. Treatment with antipsychotic drugs, such as quetiapine plus vitamin B12, have led to a decreased expression of pro-inflammatory cytokines such as IL-8, TNFα, and TGF-β, which primarily suppress immune response. These cytokines can be produced at higher levels in schizophrenic patients [261]. 

Psychotic symptoms are common in PD. Psychosis has long been considered a consequence of dopaminergic therapies used to treat the motor symptoms. However, psychotic symptoms have been linked to the intrinsic processes of the disease itself. Parkinson’s disease psychosis is one of the major challenges in the treatment of PD and includes symptoms such as visual hallucinations, passage hallucinations in the periphery of the visual fields, complex visual hallucinations, auditory, tactile, gustatory, and olfactory hallucinations, and paranoid beliefs regarding infidelity or abandonment. Such symptoms in schizophrenic patients are described as positive symptoms. However, patients with PD are also described to have negative symptoms, which in schizophrenia encompass apathy, anhedonia, flat affect, avolition, and social withdrawal [262,263].

**Figure 7:**
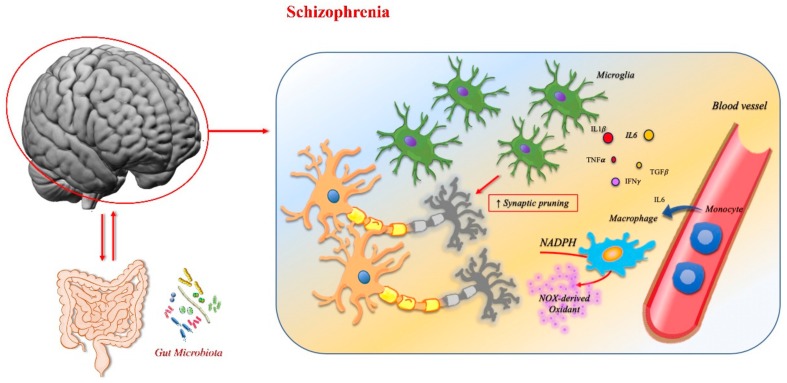
Schematic representation of the immunological mechanisms associated with schizophrenia.

In PD the activation of microglia is a common pathological finding. Microgliosis relates to the deposition in the brain of abnormal α-synuclein, which, via the toll-like receptors, can activate the production of TNFα and IL-1β by the microglial cells [264]. Interestingly, in schizophrenic patients, TNF-α shows a positive correlation with negative symptoms, whereas IL-1β has a positive correlation with positive symptoms. Figure 7 reports the main mechanisms involved in schizophrenia.

### 3.3. Post-Traumatic Stress Disorder 

**Figure 8:**
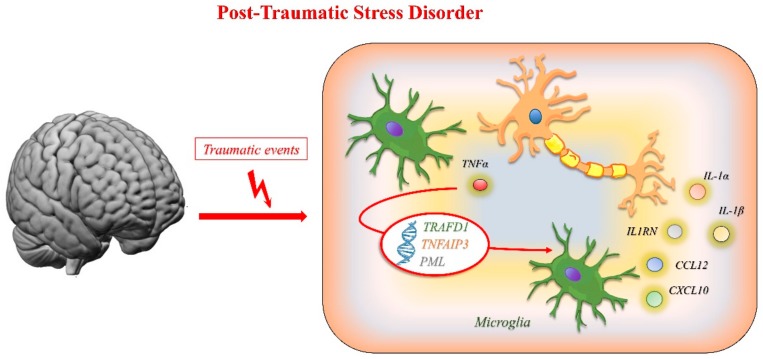
Immunological mechanisms associated with post-traumatic stress disorders.

Post-traumatic stress disorder (PTSD) may occur in individuals after experiencing terrifying events, such as traumatic incidents, war, or kidnapping. Neuroinflammation, together with sex hormones, is involved in the development of war-related PTSD [265]. Although a traumatic event triggers the activation of the hypothalamic–pituitary–adrenal (HPA) axis, with cortisol protecting from excessive or acute inflammation [266], if the stimulation is prolonged, it hesitates in an excessive inflammatory immune response [267,268]. Many molecular studies, including gene expression and epigenetics, have been carried out on PTSD. These analyses corroborated the previous findings that innate immunity could influence the progression of PTSD [269]. Genes such as *TNFAIP3*, *TRAFD1*, and *PML* are involved in the innate immune response and their expression is rapidly induced by the tumor necrosis factor (TNF). The protein encoded by *TNFAIP3* is a zinc finger protein and ubiquitin-editing enzyme, which inhibits the nuclear factor kappa-light-chain-enhancer of activated B cell (NF-kB) activation, as well as TNF-mediated apoptosis. *TRAFD1* is an LPS- and IFN-inducible gene that suppresses Toll-like receptor 4-mediated NF-kB activation. The *PML* gene product, a phosphoprotein that localizes to nuclear bodies where it functions as a transcription factor and tumor suppressor, is key to microglia activation and the production of key inflammatory cytokines, such as IL-1α, IL-1β, IL-1RN, CXCL10, CCL12, and TNFα [270]. Figure 8 reports the main mechanisms involved in post-traumatic stress disorders.

### 3.4. Autism Spectrum Disorder

The term autism spectrum disorder (ASD) refers to a group of pervasive neurodevelopmental disorders that involve moderately to severely disrupted functioning with regard to social skills and socialization, expressive and receptive communication, and repetitive or stereotyped behaviors and interests [271]. Boys are diagnosed with ASD four to five times more frequently than girls, but the cause of this male prevalence remains obscure. Studies on autism genetics highlight the role of small de novo copy number variations in genes associated with synaptic transmission, transcriptional regulation, or epigenetic modifications of the genome, but there is no clear gender bias in the identified risk genes [272]. Other studies look at ASD as a classic complex trait in which heritability is framed as a predisposition, based on the cooperation of multiple common genetic variants rather than the drastic effect of a few major genes [273,274]. 

On the other hand, the involvement of environmental factors is assumed to have the main impact on pathogenesis, which would explain the fairly rapid recent increase in ASD prevalence, which cannot be attributed to genetic changes.

This increase in ASD has been paralleled by the epidemic increase in atopic disorders in childhood in the general population [275,276,277]. Dysfunctions of the immune system may therefore bridge the action of environmental changes in interfering with neurodevelopment. In this scenario, mast cells may play a crucial role, given their readiness to be activated by many entities, including microbes, heavy metals, food, and stress. This has been outlined a model in which prolonged mast cell triggering led to the production of a wide range of immunomodulators that alter the functionality of BBB and cause a microglia activation. Microglia activation, in turn, may create an atmosphere with a dwelt, altered cytokine profile, inadequate to ensure neuronal pruning and connectivity assembly in crucial life stages for neurodevelopment [278,279]. Many cytokines, including IL-1B, IL-6, IL-4, IFN-γ, and TGF-B, can influence the function and development of the nervous system, as they share signal pathways with neurotrophic factors [280]. A well conducted Norwegian study reported that exposure to prenatal fever, especially during the second trimester of gestation, is associated with an increased risk of developing ASD in the unborn child [281]. In the same study the authors found that antipyretic therapy can mitigate the risk in all the periods analyzed. 

The innate immune system and inflammatory mediators may contribute to directing brain masculinization. Indeed, neuroanatomical sex differences are established early, beginning in the uterus and extending to the postnatal period. A major driver is an increase in androgens and estrogens in the brain of developing males as a result of steroidogenesis by the fetal testis. In rodents, normal masculinization of some brain regions involves inflammatory signaling molecules, such as prostaglandins, which can be derived from activated microglia. Inflammation during pregnancy in humans may be a risk factor for the development of ASD, with evidence that the greater the inflammation, the more severe the disorder [282]. 

**Figure 9:**
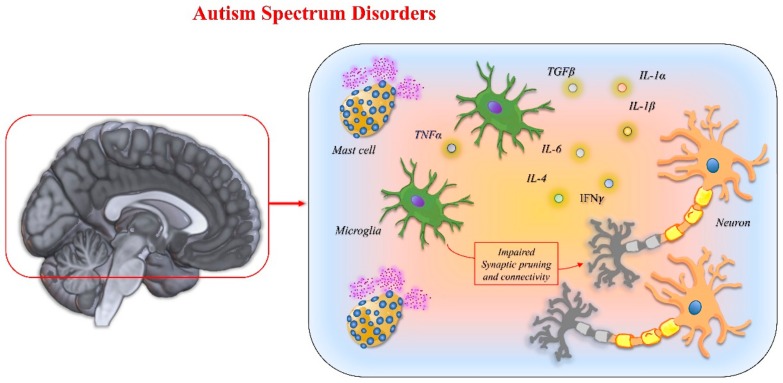
The main immunological mechanisms associated with autism spectrum disorders.

TNFα has recently been described as a key molecule dysregulated in ASD children [283]. In an animal model, it was shown that a neonatal immune challenge with LPS triggered a series of long-lasting behavioral and immune/neurotrophic alterations, thus revealing the effect of a dysregulated innate immune system on the developing brain [284]. This is in line with findings in humans [285]. Figure 9 reports the main mechanisms involved in autism spectrum disorders.

## 4. Differences and Similarities between Neurological and Psychiatric Disorders

The extent to which the classical differentiation between neurological and psychiatric disorders expresses a biologically defined distinction is still an open issue. Both classes of disorders are related to brain dysfunctions. However, one of the most difficult problems is to understand why dysfunctions within the same organ may cause either neurological or psychiatric diseases. Neurological disorders are considered “organic” brain diseases because they produce symptoms due to evident damage in specific regions of the nervous system. On the other hand, psychiatric disorders are characterized by a disturbed behavior and emotional state as a “functional” effect of brain impairment. 

However, the line between these disorders is grey. It is well known that affective or psychotic symptoms typical of psychiatric disorders can occur in neurological diseases [286,287], whereas psychiatric disorders can produce motor or sensitivity symptoms commonly observed in neurological diseases [288]. Brain imaging studies, which open up an in vivo window into the brain, have also revealed that both neurological and psychiatric diseases are characterized by the occurrence of shared neuroanatomical and neurofunctional alterations, although with some differences [289].

Although there are causal interconnections between the immune response and brain structure and function, understanding exactly how the immune system impacts brain illness is very problematic, due to the unique and distinctive properties of the CNS. The CNS tissue is different from other peripheral tissues in terms of its regional complexity, connection and network properties, specific metabolic demands, and the interplay with BBB that filters the exposure to pathogens. The result is an organ with mechanisms and potential still to be discovered.

Biological evidence from in vitro and in vivo studies on neuroinflammation and neuro-immunity has confirmed the presence of common immune-mediated mechanisms underpinning both these disease classes, although with some differences:

(i) Response mechanisms of innate immunity. The overall response is similar for both disorders and is characterized by inflammation mediated by resident glial cells, which produce molecules which, in turn, facilitate the recruitment of other immune cells, including the peripheral innate immunity cells (Figure 2, Figure 3, Figure 4, Figure 5, Figure 6, Figure 7, Figure 8 and Figure 9). These soluble inflammatory molecules include cytokines, chemokines, and complement proteins, with a profile that presents subtle differences among these disorders. Table 1 reports the main cytokines and chemokines involved in neurological and psychiatric diseases.
(ii) Triggering of response and toxicity. Neurons are characterized by a notable susceptibility to inflammatory thrust, like that produced by cytokines, inducible nitric oxide synthase (iNOS), and phagocytic NADPH oxidase in the brain. If uncontrolled, these stimuli cause neuronal death via oxidative damage. The neurotoxicity is clearly more evident in neurological diseases, especially in neurodegenerative ones. Indeed, unlike psychiatric disorders, neurodegenerative diseases are characterized by severe, inexorable neuronal loss, and lead to the atrophy of specific brain regions. A particular characteristic of neurodegenerative diseases is the accumulation of misfolded proteins that contribute to neurotoxicity and in themselves are an inflammatory stimulus that can trigger and amplify the immune response.
(iii) Gene/environment interaction and age of onset. Recent evidence has revealed that both neurological and psychiatric disorders share mechanisms of individual predisposition to the development of the disease. Despite this, there are differences in the weight of the genetic component between these disorders. Some neurodegenerative diseases are directly caused by a genetic mutation (such as HD, or some forms of AD, PD, FTD, and ALS). In other cases, the genetic component represents a risk factor that interacts with environmental factors, but the brain compensatory abilities are such that, despite the degenerative process beginning many years earlier, the disease becomes evident in senile or pre-senile age. In psychiatric diseases, the genetic susceptibility component always needs to fit together with environmental factors, sometimes represented by intercurrent infections, toxic environmental substances, or other insults that stimulate the inflammatory response in a period in which the correct brain architecture is being built (childhood or even intrauterine life), giving rise to neurodevelopmental disorders. In addition, there is a widespread genetic overlap across psychiatric disorders. In contrast, neurological disorders seem to be genetically distinct from each another and from psychiatric disorders, thus suggesting that genetic influence is not similar among these disorders. Differences in the timing and means of gene–environment interactions, as well as differences in interactions between genes, can produce clinical peculiarities of these [290]. The lack of large-scale genetic variants shared between neurological and psychiatric diseases could underpin the main etiological and pathogenetic differences, and be combined with neuroinflammatory mechanisms in these disorders.
(iv) Gut microbiota–brain axis. The complex and multifaceted crosstalk between the microbiota, immune system, and CNS is a fundamental emerging pathophysiological aspect shared by both psychiatric and neurodegenerative disorders. Growing evidence suggests that brain-resident and peripheral immune cells play a pivotal role in managing gut microbiota–brain interaction. The gut microbiota is a critical factor in modulating the activities of glial cells resident in the brain, which are essential for several key processes, such as neurogenesis, neural growth, synapse homeostasis, neurotransmission, CNS immune response, and BBB integrity. The microbiota also activates peripheral immune response, with critical effects on brain inflammation. Accordingly, the interaction between gut microbiota and the immune system is a key factor in the pathogenetic cascade, leading to neurodevelopmental, psychiatric, and neurodegenerative diseases.
(v) Therapeutic perspective. The therapeutic strategies currently adopted in neurological and psychiatric disorders are symptomatic and aim to restore the altered neurotransmitter balance or to provide depleted mediators. In psychiatry, they have led to the improvement of some disorders, and have made severe conditions, such as schizophrenia, more manageable. In the field of degenerative diseases, although therapeutic strategies are useful for counteracting the main symptoms, they do not lead to effective action on the course of the disease. Further knowledge on the immunological mechanisms underlying these pathologies may lead to common therapeutic strategies, aimed at modulating the inflammatory response underlying these disorders.

## 5. Discussion

The close relationship between innate immunity and brain diseases is raising interest across the wide realm of neurodegenerative and neuropsychiatric disorders. 

The central nervous system has historically been considered as a privileged place regarding immunity response. On the other hand, neurological and psychiatric diseases have traditionally been considered as opposite conditions, with few analogies from a pathogenetic point of view. 

The evidence addressed in the present review questions these assumptions and supports new insights into the role of neuroinflammatory processes in the brain and their contribution in both neurological and psychiatric diseases. It has now become clear that innate immunity and, in particular, glial cells, have a key function not only in the resilience of the central nervous system, but also in surveys of local microenvironment and synaptic pruning during brain development. Consequently, perturbations in the subtle balance across immune cells, neurons, and glial cells, essential for nervous system efficiency, may impact large-scale brain functioning occurring in neurodegenerative and psychiatric disorders. 

**Figure 10:**
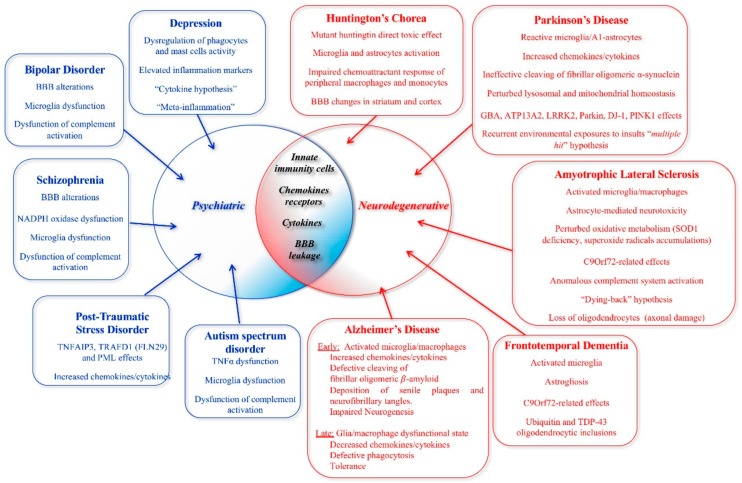
The pathophysiological effects of innate immune dysregulation leading to neuropsychiatric and neurodegenerative diseases.

There are clearly significant similarities between these conditions in terms of their relationship with innate immunity (Figure 10). Chronic inflammation is a shared characteristic of both disorders, and high activation of resident glial cells (i.e., microglia), with the release of proinflammatory soluble mediators, has been found in neurodegenerative and psychiatric diseases. Several genes associated with these conditions have been reported to perturb the microglial function, either by impairing phagocytic capacity and altering synaptic pruning, or by favoring protein aggregation and degradation deficits, thus further triggering proinflammatory pathways. Moreover, in both conditions there is a deep and complex interaction between gut microbiota and the nervous system through a regulatory action of innate immunity. This suggests that the altered role of innate immunity represents a common denominator between neurological and psychiatric disorders, which could be considered pathogenically related. Exactly why analogous pathogenic processes cause different disorders belonging to different nosological entities is still an open issue, as is whether the immune response should be considered a cause or a consequence of the pathological process. 

Nonetheless, these findings have great potential in terms of new therapeutic approaches to these diseases and the clinical management of patients. Indeed, this new knowledge about the role of innate immunity in neurological and neuropsychiatric diseases pathogenesis may help to identify new potential therapeutic targets for the treatment of these disorders by manipulating particular aspects of the innate immune response. Thus, specific approaches, such as cell depletion therapies, tolerance-inducing strategies, vaccinations, and the use of stem cells, have been implemented, with differing results [291]. Although current therapeutic approaches point at modulating neuroinflammation during the manifest stages of the diseases, future strategies should aim, on the one hand, at disease prevention, and, on the other hand, at personalized therapy based on the patient’s individual profile. 

Prompt and specific diagnosis as well as suitable parameters for checking disease progression and the response to therapy are therefore fundamental. 

Progress in biomarker research is essential in order to facilitate early diagnosis and efficient stratification in clinical trials, and to accelerate new therapeutic target identification and drug experimentation. Structural and functional markers of BBB impairment, axonal/neuronal injury, and demyelination, as observed throughout neuroimaging approaches, could represent helpful biomarkers of neuroimmunological diseases. In addition, the soluble markers of altered immune response should be considered, such as cytokines, chemokines, antibodies, and changes in cellular subpopulations [292]. This could be achieved by looking for new biomarkers that are specific, minimally invasive, and which reflect the course of the disease and the individual response to therapy. 

In conclusion, deciphering the complexity of neuroimmune crosstalk has a strong impact in terms of understanding brain diseases. Revealing the processes by which common mechanisms in the brain can lead to different disorders represents one of the greatest challenges of basic and translational neurosciences: the aim being to identify early biomarkers of the disease as well as new potential targets for drug development.

## Figures and Tables

**Table 1:** List of cytockines and chemokines dysregulated and disease involvement.

Abbreviation	Cytokine or Chemokine	Main Functions of Cytokine	Cytokine Source	Pathology [References]
IFNα	Interferon alpha	↑ NK cells and CTL functions Influence isotype switching	Activate macrophages, monocytes	AD [10] Depression [203]
IFNγ	Interferon gamma	↑ NK cells and CTL functions ↑ APC production of IL-12 ↓ IL-4 production Influence isotype switching	Activate macrophages	PD [55] Schizophrenia [249]
IL-1	Interleukin-1	Pro-inflammatory ↑ Acute phase response	Macrophages, neutrophils, epithelial and endothelial cells	AD [10] PD [90] Depression [200] Schizophrenia [249]
IL-2	Interleukin-2	Th1 cytokine ↑ T and B cell activation ↑ NK cell proliferation and production of TNF, IFNγ Required for Treg cell differentiation	Activated T-cells	Depression [199]
Il-6	Interleukin-6	Pro-inflammatory ↑ Acute phase response	Activated phagocytes, Endothelial cells, Some activated T cells	AD [10] PD [244] Depression [201] Schizophrenia [249] PTSD [267]
IL-8	Interleukin-8	CXC chemokine ↑ Neutrophil chemotaxis and degranulation	All cell types encountering TNF, IL-1 or bacterial endotoxin	Schizophrenia [261]
IL-12	Interleukin-12	Required for Th1 cell differentiation ↑ production of IFN γ by macrophages, activated Th1 cells, NK cells ↑ DC and macrophage cytokines secretion ↑ CTL and NK cytotoxicity Influences isotype switching	Activated macrophages, Dentritic cells, neutrophils, monocytes, B cells	Schizophrenia [249]
TNF	Tumor Necrosis Factor	Potent inflammatory, immunoregulatory, cytotoxic, antiviral, pro-coagulatory, and growth stimulatory effects	Many types of activated hematopoietic and non-hematopoietic cells	AD [10] PD [264] Depression [203,204] Schizophrenia [249] PTSD [270] ASD [283]
TGFβ	Transforming growth factor-β	Anti-inflammatory, immunosuppressive	Most activated hematopoietic cells; some non-hematopoietic cells	AD [10] Schizophrenia [249]
CXCL10 (IP-10)	C-X-C motif chemokine 10 (Interferon gamma-induced protein 10)	Chemoattractant for monocytes, macrophages, T cells, NK cells, and DC	Several cell types in response to IFN-γ: Monocytes, endothelial cells and fibroblasts	PTSD [270]
CXCL12 (SDF-1)	C-X-C motif chemokine 12 (stromal cell-derived factor 1)	Chemoattractant for lymphocytes, macrophages, hematopoietic cells. In neuroinflammation attraction of leukocytes across the blood brain barrier.	Leucocytes, endothelial cells, glial cells and neurons.	PD [55]
CCL12	Chemokine (C-C motif) ligand 12	Chemoattractant for eosinophils, monocytes and lymphocytes.	Leucocytes, endothelial cells and fibroblasts	PTSD [270]

## Abbreviations

AD	Alzheimer’s disease
ALS	Amyotrophic lateral sclerosis
BBB	Blood-brain barrier
CNS	Central nervous system
CRP	C-reactive protein
CD	Crohn’s disease
CXCL	“C-X-C” motif chemokine ligand
CXCR	“C-X-C” motif chemokine receptor
DAMP	Damage associated molecular pattern
DC	Dendritic cell
DPR	Dipeptide repeats
FTD	Frontotemporal dementia
GWA	Genome-wide association studies
HD	Huntington’s disease
HLA-DR	Human leukocyte antigen – DR isotype
IL	Interleukin
IL-1RN	Interleukin 1 receptor antagonist protein
IFN	Interferon
iPSC	Induced pluripotent stem cell
IBD	Inflammatory bowel disease
LE	Limbic encephalitis
MBL	Mannan-binding lectin
MND	Motor neuron disease
NK	Natural killer
PAMP	Pathogen associated molecular pattern
PD	Parkinson’s disease
PRRs	Pattern-recognition receptors
ROS	Reactive oxygen species
TNF	Tumor necrosis factor
TGF	Transforming growth factor
UC	Ulcerative colitis
